# Virtual care for young adults with type 1 diabetes: a 12-month follow-up of a wait-list randomized controlled trial

**DOI:** 10.1186/s12902-026-02371-2

**Published:** 2026-06-23

**Authors:** Rebecka Husdal, Eva Toft, Jan W. Eriksson, Johan Fischier, Andreas Rosenblad, Elisabet Nerpin, Janeth Leksell

**Affiliations:** 1 Department of Medical Sciences, Clinical Diabetology and Metabolism, Uppsala University, Akademiska Sjukhuset, Uppsala, SE-75185 Sweden; 2 Department of Clinical Science and Education at Södersjukhuset, Karolinska Institutet, Stockholm, Sweden; 3 Diabetes Unit, Ersta Hospital, Stockholm, Sweden; 4 Department of Neurobiology, Care Sciences and Society, Division of Family Medicine and Primary Care, Karolinska Institutet, Stockholm, Sweden; 5 Department of Statistics, Uppsala University, Uppsala, Sweden; 6 School of Health and Welfare, Dalarna University, Falun, Sweden; 7 Department of Medical Sciences, Respiratory Medicine, Allergy and Sleep, Uppsala University, Uppsala, Sweden; 8 Department of Medical Sciences, Clinical Physiology, Uppsala University, Uppsala, Sweden

**Keywords:** Glycaemic control, Quality of life, Treatment satisfaction, Type 1 diabetes, Virtual diabetes clinic, Young adults

## Abstract

**Background:**

Digital tools are increasingly used in diabetes care, but their long-term effectiveness remains uncertain. A previous randomized controlled trial (RCT) demonstrated promising effects on perceived burden and quality of life after 6 months. The present study extends these findings through a 12-month follow-up, aiming to evaluate the long-term effects of virtual care on glycaemic control, treatment satisfaction, and quality of life among young adults with type 1 diabetes.

**Methods:**

This single-centre wait-list RCT included 70 young adults aged 18–25 years with type 1 diabetes in Stockholm, Sweden. Participants randomized to the intervention group had access to the virtual platform for 12 months whereas participants in the wait-list control group received access after 6 months. The platform facilitated real-time communication with healthcare providers. Glycated haemoglobin (HbA1c) levels, time in range, time below range, diabetes treatment satisfaction, and quality of life were assessed at baseline and at the 6- and 12-month follow-ups. Comparisons within and between groups were performed using Welch’s paired and independent samples t-test, the Wilcoxon signed rank test, and the Mann-Whitney test.

**Results:**

At the 12-month follow-up, the intervention group showed a decreased HbA1c level from baseline (62.8 to 58.6 mmol/mol; P = 0.051) and improved time in range (46.6% to 56.9%; P = 0.002). In the wait-list control group, social burden decreased significantly from baseline to 12 months (4.3 to 0.03; P = 0.030). Other measures of quality of life, including social and emotional health, showed no significant differences.

**Conclusions:**

Virtual diabetes care may enhance engagement and modestly improve glycaemic control in young adults, serving as a practical complement to standard outpatient follow-up.

**Trial registration:**

ISRCTN (ref: 73435627, registration date: 23/10/2019): https://doi.org/10.1186/ISRCTN73435627.

## Background

Young adults with type 1 diabetes stand at a crossroads between independence and lifelong self-care [1]. As they move from paediatric to adult healthcare during the developmental stage of emerging adulthood, they are expected to manage complex diabetes treatment routines while simultaneously navigating education, work, and social transitions [2]. This period of emerging adulthood is characterized by new freedoms but also increased vulnerability [3], as self-care demands intensify and regular clinic-based follow-up and parental support often decrease [4, 5]. Despite the complexity of self-care in type 1 diabetes, a systematic review found that psychological treatments have limited impact on glycated haemoglobin (HbA1c), challenging the common assumption that such interventions should lead to clear improvements in achieving blood glucose targets [6]. However, several virtual interventions have demonstrated improvements in diabetes-related distress, self-efficacy, and quality of life among young adults, even in the absence of significant glycemic effects [7–9].

In Sweden, the incidence of type 1 diabetes has increased over time towards younger age at onset [10], requiring continuous monitoring of blood glucose levels, insulin administration, and lifestyle adjustment to prevent acute and long-term complications [11]. As these individuals reach adulthood, the transition to adult care which in Sweden typically occurs around 18 years of age, entails a substantial increase in personal responsibility for self-care. Many emerging adults report feeling insufficiently prepared or inadequately supported during this transition [12]. Consequently, there is a growing need for care models that are both flexible and responsive to the realities of this developmental stage [13, 14].

At the same time, this generation has grown up with constant digital access. Smartphones, continuous glucose monitors (CGMs), and cloud-connected insulin pumps have transformed how individuals interact with their data, with healthcare professionals, and with their peers [15]. Today, nearly all (92%) young Swedish adults with type 1 diabetes use CGM technology [16] enabling real-time glucose tracking and data sharing, which is a development that naturally paves the way towards additional technology-assisted care.

However, while digital tools are increasingly integrated into diabetes management, their effectiveness in improving long-term outcomes remains uncertain. Meta-analyses suggest that mobile apps alone may not significantly reduce HbA1c levels [17], while studies of video consultations [18] have shown mixed results, with some improvements in satisfaction and quality of life, particularly during the COVID-19 pandemic. Despite growing interest, there is still limited evidence on whether structured virtual care can sustainably complement standard care and enhance both clinical and patient-reported outcomes among young adults in emerging adulthood.

In response to these challenges, we developed and implemented a virtual clinic for young adults with type 1 diabetes in Sweden, using the secure Vista Dialog platform to enable video meetings and chat communication with diabetes specialists [19]. A previous 6-month follow-up demonstrated promising effects on perceived burden and quality of life [20]. In line with this, previous studies also indicate that virtual interventions may improve engagement and psychosocial outcomes [21, 22]. However, there is still limited evidence on their long-term effects on both clinical and patient-reported outcomes in young adults. The present study extends these findings through a 12-month follow-up randomized wait-list controlled trial, aiming to evaluate the long-term impacts of virtual care on glycaemic control, treatment satisfaction, and quality of life.

### Study aims

This study aimed to evaluate the effects of a virtual diabetes clinic on glycaemic control, treatment satisfaction, and quality of life in young adults with type 1 diabetes at 12 months and across the 6–12-month follow-up period.

## Methods

### Study design

A detailed description of the study design and methods is given in the previously published study protocol [19]. Briefly, in this single-centre prospective randomized (balanced 1:1) wait-list controlled trial, individuals with type 1 diabetes were recruited from January 2019 (baseline) and followed-up to November 2022 (12-month follow-up). The trial was registered in ISRCTN on 23 October 2019 (ref: 73435627): 10.1186/ISRCTN73435627.

### Recruitment of participants and randomization

All participants had type 1 diabetes and were recruited from a single hospital in Stockholm, Sweden by the healthcare professionals using the diabetes clinic’s register. The inclusion criteria were having access to a smartphone or computer, having a diabetes duration of more than 1 year, being 18–25 years old and the use of a CGM. Individuals were not eligible to participate if they had a diagnosis of severe depression, eating disorder or other severe mental illness, a history of alcohol and/or drug abuse, or current severe micro and/or macrovascular complications related to diabetes. It should be noted that participant recruitment took place during the COVID-19 pandemic, which affected the ability to reach the intended sample size of *n* = 100. Participants were recruited by clinic nurses and randomly assigned to either the intervention or wait-list control group after providing their informed consent. Randomization was conducted using sealed envelopes containing randomized cards prepared by an independent individual not involved in the inclusion or care of the patients. The envelopes were numbered 1 to 100. The staff member recruiting the patient took the envelope that was on top of the stack. Clinic nurses were responsible for inclusion, randomization and data collection. All clinic staff received training in study procedures and data collection. All study materials were coded and securely stored. More detailed information on the randomization procedure is provided elsewhere [20].

### Data collection

**Fig. 1:**
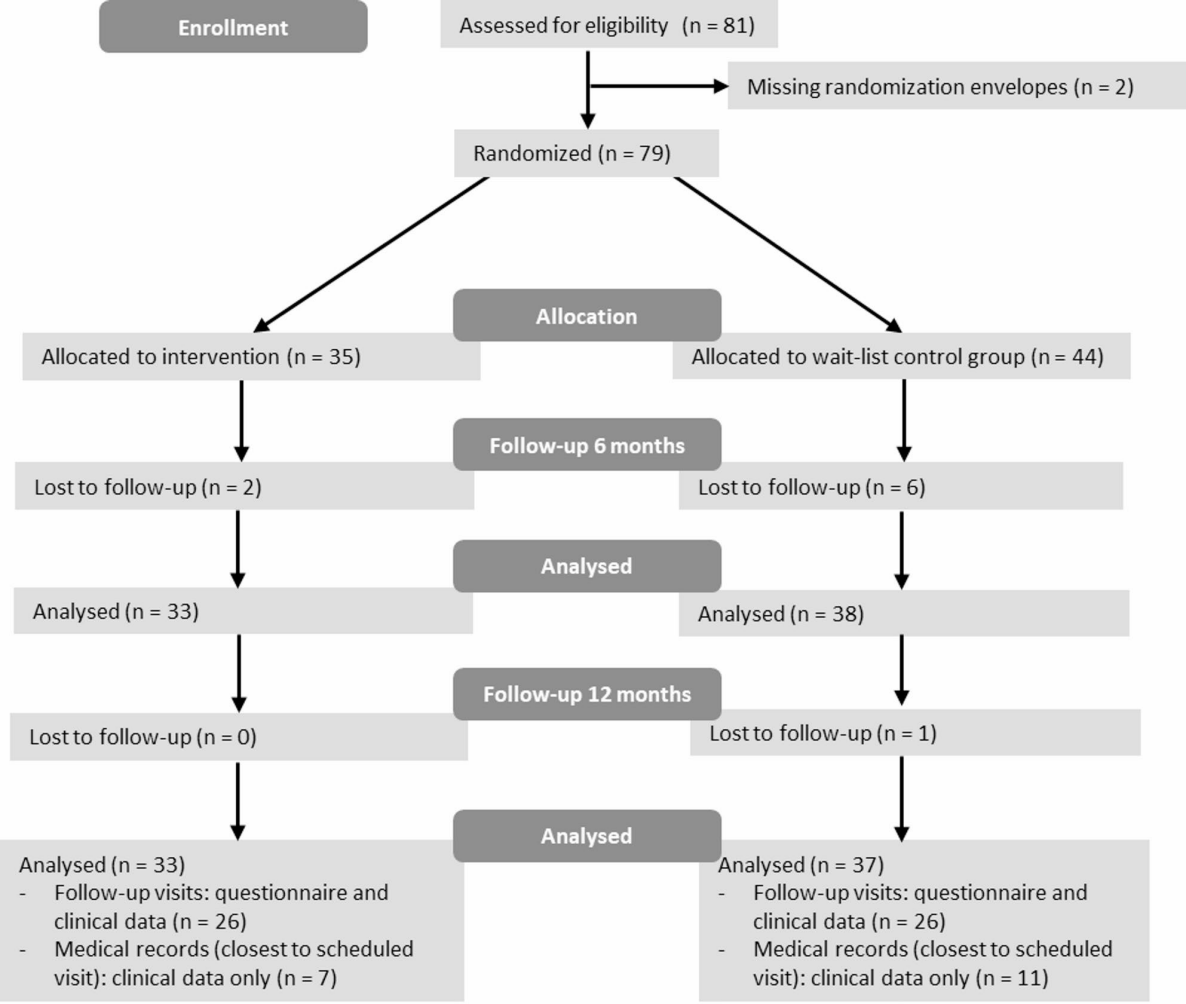
Flowchart of the trial from baseline to the 12-month follow-up

Of the 79 participants who were recruited at baseline, 71 were followed up at 6 months. At the 12-month assessment, 70 participants remained in the study: 33 assigned to the intervention group and 37 to the wait-list control group (Fig. 1). Information on loss to follow-up at 6 months is reported in a previous article [20]. At the 12-month follow-up, one participant from the wait-list control group was excluded because of missing data due to the absence of scheduled visits to the diabetes clinic between November 2020 and June 2022.

Data collection for both clinical and psychometric measures was conducted during routine outpatient care at the diabetes clinic. Usual care at the study site remained predominantly in-person throughout the study period, including during the COVID-19 pandemic. Baseline assessments were performed prior to the intervention, and outcome measurements were collected at the baseline, 6-month, and 12-month time points. Background characteristics such as sociodemographic data including age, sex, living situation, and education were collected at baseline. Clinical data were collected at all time points, including HbA1c, time in range (TIR), and time below range (TBR). Information on diabetes duration, treatment type (multiple daily injections or continuous subcutaneous insulin infusion), and body mass index was gathered to provide a comprehensive participant profile. For 18 of the 70 participants (7 intervention, 11 wait-list control), clinical data for the 12-month follow-up were obtained from medical records at the time point closest to the scheduled follow-up after the intervention had ended. No psychometric data were available for these participants.

### Intervention

The intervention group was granted immediate access to the virtual care platform after baseline assessment. The intervention utilized Vista Dialog, a secure virtual care platform accessed via a smartphone app for participants and via a web portal for healthcare professionals, enabling real-time communication through chat, video calls, and appointment scheduling with diabetes specialist nurses or doctors. The platform complemented the usual care provided at the outpatient clinic. In addition to the scheduled in-person visits, participants had the option to book online appointments and initiate spontaneous video calls with a nurse if needed. Participants could also upload insulin pump and CGM data for review, allowing them to proactively communicate their needs as they arose. Further details of the intervention are provided in the published study protocol [19]. The wait-list control group received care as usual from inclusion to the 6-month assessment, after which they were granted access to the virtual care platform in the same way as the intervention group.

### Primary and secondary outcomes

The primary outcome of the 12-month follow-up was glycaemic control. During routine clinical appointments, the following clinical outcomes were collected: HbA1c (mmol/mol; Afinion™ 2, Abbott, USA), reflecting average plasma glucose levels over the preceding 8–12 weeks; TIR, defined as the percentage of time glucose levels were within the target range (3.9–10 mmol/L); and TBR, defined as the percentage of time glucose levels were < 3.9 mmol/L, indicating hypoglycaemia. Real-time CGM and (rtCGM) / intermittently scanned CGM (isCGM) were used to measure TIR and TBR. Insulin dosage was extracted and recorded through the diabetes management software DIASEND^®^; daily insulin dosage was collected where possible.

A secondary outcome was diabetes treatment satisfaction, assessed using the Diabetes Treatment Satisfaction Questionnaire (DTSQ) [23]. The DTSQ questionnaire is composed of three areas with a total of eight questions. The first area includes six questions covering aspects of treatment satisfaction such as satisfaction with current treatment, flexibility, convenience, understanding of diabetes, willingness to recommend treatment to others, and willingness to continue. These items are rated on a 7-point Likert scale (0–6 points), with higher scores indicating greater satisfaction, resulting in a total score of 0–36 points. The second and third area consists of single questions each related to assess perceived frequency of hyperglycaemia and hypoglycaemia during the preceding weeks, also rated on a 7-point Likert scale (0–6 points).

Another secondary outcome was perceived health and quality of life, assessed using the validated Check Your Health questionnaire [24]. ‘Check your health’ measures perceived physical and emotional health, social relationships, and general HRQoL on four vertical thermometer scales ranging from 0 to 100, where 0 indicates the lowest perceived health and HRQoL. For each domain, participants rate both their current status with diabetes and how they believe it would be without diabetes on the same scale. Diabetes-related burden is defined as the difference between these two ratings. For example, the difference between perceived physical health with diabetes and perceived physical health without diabetes is defined as the physical burden of diabetes. The cut-off values for no burden (0), low burden (1–10), high burden (11–29), and very high burden (> 30) are arbitrary.

### Ethics

Informed consent was obtained from all enrolled participants according to the principles and amendments of the Declaration of Helsinki. The study was approved by the Swedish Ethical Review Authority in Uppsala (refs: 2018–568 and 2019–00133).

### Statistical analysis

Categorical data are presented as frequencies and percentages, while ordinal and continuous data are presented as mean values with accompanying standard deviations (SDs). Tests of differences between paired observations within groups (i.e., either the intervention or the wait-list control group) were performed using Welch’s paired *t*-test for continuous data and the Wilcoxon signed rank test for ordinal data. Paired observations refer to measurements taken from the same participants at two different time points. For differences between (non-paired) groups (i.e., when comparing the intervention and wait-list control group), Welch’s independent samples *t*-test was used for continuous data and the Mann-Whitney test for ordinal data. Non-paired observations included all participants who contributed data at a specific time point, allowing all available observations to be included in the analysis. Missing data were handled using listwise deletion. Statistical analyses were performed using R version 4.5.0 or higher (R Foundation for Statistical Computing, Vienna, Austria), with two-sided *P*-values < 0.05 considered statistically significant.

## Results

At baseline (Table 1), the intervention and wait-list control groups were well balanced with respect to demographic and clinical characteristics. The proportion of women was slightly lower in the intervention group (60.0%) compared with the wait-list control group (70.5%). The mean (SD) diabetes duration was similar between the groups: 10.7 (4.7) years in the intervention group and 10.5 (5.1) years in the wait-list control group. The mean (SD) age was 19.8 (1.8) years in the intervention group and 20.7 (1.9) years in the wait-list control group. Participants in the intervention group had a slightly higher mean (SD) HbA1c of 62.8 (14.8) mmol/mol, compared with 58.8 (11.5) mmol/mol in the wait-list control group. Insulin treatment modality was comparable, with more than half of participants in both groups using insulin pumps and the remainder using multiple daily injections. Use of glucose monitoring technologies was also similar in the two groups, with most participants using intermittently scanned CGM (61%), followed by real-time CGM (36%), and only a few using capillary glucose monitoring (3%). No significant baseline differences were observed between groups, except for educational level, which showed a skewed distribution.

**Table 1:** Baseline characteristics of the intervention group and wait-list control group

Variable	Intervention	Wait-list control group
	*n* = 35	*n* = 44
Female sex, n (%)	21 (60.0)	31 (70.5)
Age (years), mean (SD)	19.8 (1.8)	20.7 (1.9)
Years with diabetes, mean (SD)	10.7 (4.7)	10.5 (5.1)
HbA1c (mmol/mol), mean (SD)	62.8 (14.8)	58.8 (11.5)
Time in range (%), mean (SD)	45.9 (13.3)	49.7 (16.5)
Glucose monitoring, n (%)		
- rtCGM	10 (29)	18 (41)
- isCGM	23 (68)	25 (57)
- Hand-held glucometer	1 (3)	1 (2)
Pump, n (%)	18 (51)	22 (50)
Multiple doses, n (%)	16 (49)	22 (50)
Education level, n (%)		
- Primary	11 (31.4)	5 (11.4)
- Secondary	22 (62.9)	35 (79.5)
- College/university	2 (5.7)	4 (9.1)

Note: CGM, continuous glucose monitor; isCGM, intermittently scanned CGM; rtCGM, real-time CGM; SD, standard deviation. Missing values: glucose monitoring, intervention group *n* = 1; time in range, intervention group *n* = 2, control group *n* = 1

### Usage of virtual care in the intervention group and wait-list control group after 12 months

**Fig. 2:**
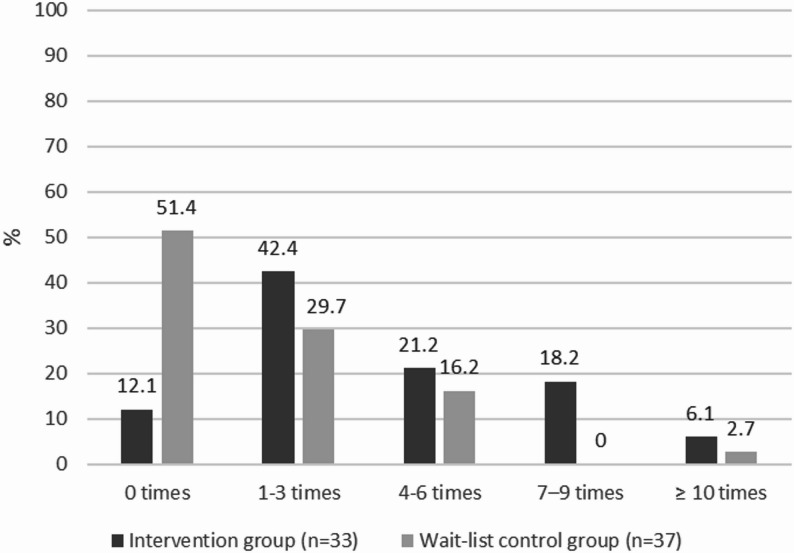
Usage of the chat function in the virtual care platform, divided by study group: intervention (*n* = 33) and wait-list control (*n* = 37)

**Fig. 3:**
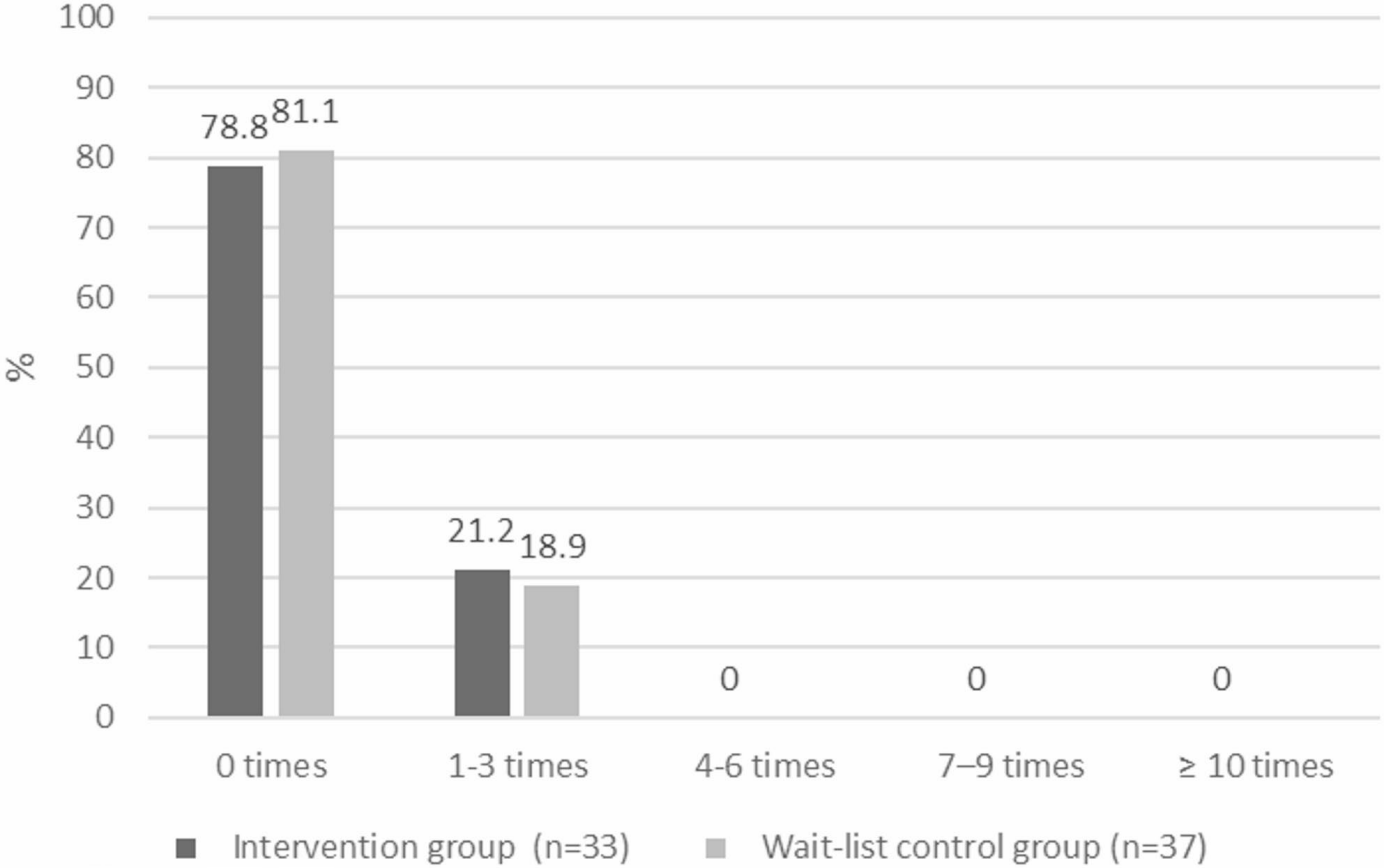
Usage of the video meeting function in the virtual care platform, divided by study group: intervention (*n* = 33) and wait-list group (*n* = 37)

Of the 79 participants included at baseline, 70 completed the 12-month follow-up (33 in the intervention group and 37 in the wait-list control group). At the 6-month follow-up, the wait-list control group was granted full access to the virtual care platform. Hence by the 12-month follow-up, all participants had access; at this point, 3 (9.1%) individuals in the intervention group and 19 (51.4%) in the wait-list group did not use the virtual diabetes care. Chat was the most commonly used communication path in both groups, with 14 (42.4%) individuals in the intervention group and 11 (29.7%) individuals in the wait-list control group using it 1–3 times. About one fifth (21.2%) of the intervention group used the chat function at least 4–6 times, compared to 16.2% in the wait-list control group (Fig. 2). The use of videoconferencing was less common, with only 7 (21.2%) individuals in the intervention group and 7 (18.9%) in the wait-list control group reporting having used it 1–3 times (Fig. 3).

### Glycaemic control

**Fig. 4:**
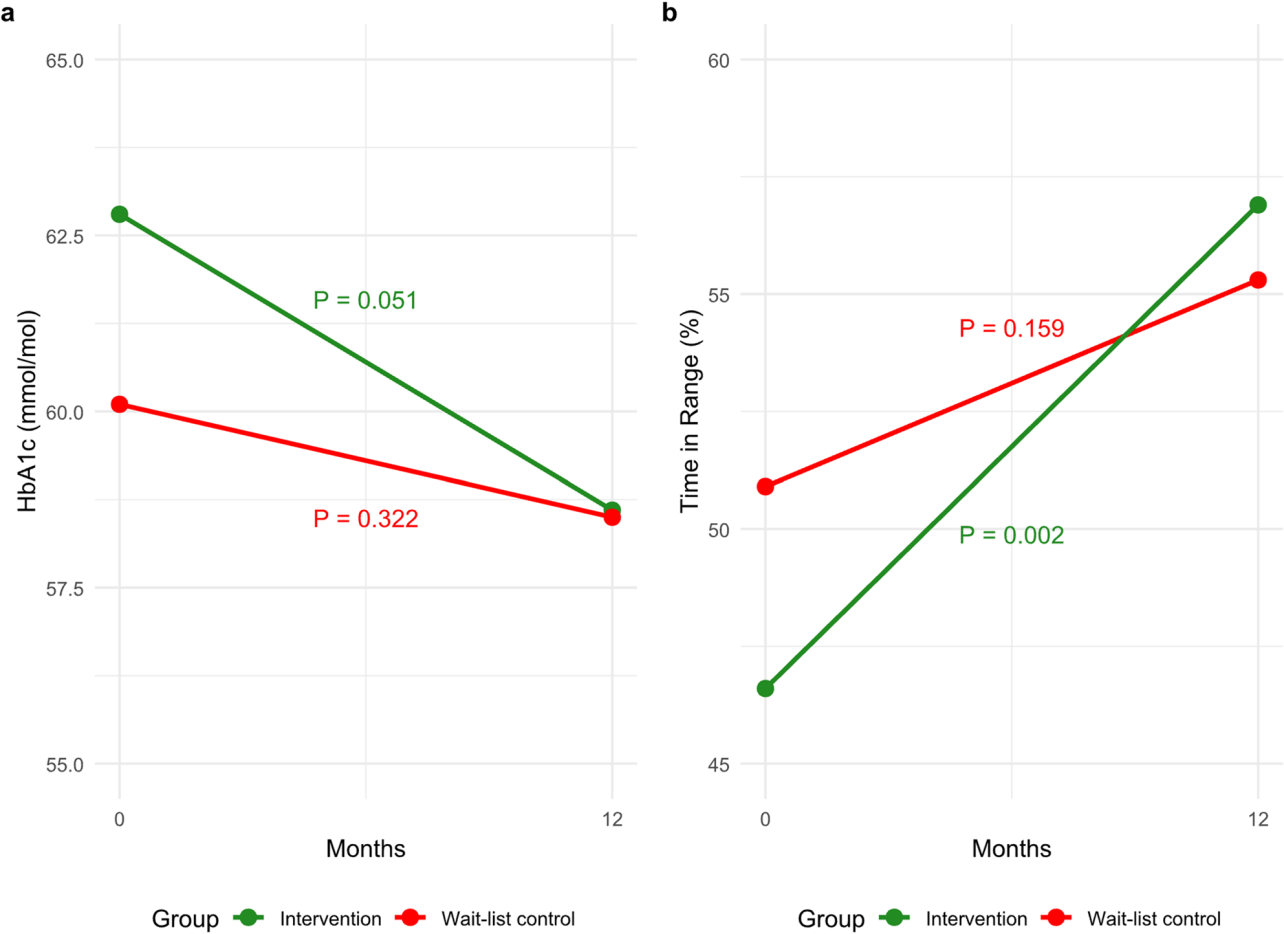
Change in (**a**) HbA1c (mmol/mol) and (**b**) time in range (%) for participants with measurements at both baseline (0 months) and the 12-month follow-up within the intervention and wait-list control groups, respectively. *P*-values are calculated for within-group changes

The results at the 12-month follow-up should be interpreted in the light of differences in exposure to the intervention: the intervention group had access to the virtual platform throughout the study period, whereas the wait-list control group had access for only the final 6 months. In the intervention group, HbA1c decreased from baseline to 12 months (62.8 to 58.6 mmol/mol); this change approached statistical significance (*P* = 0.051; Fig. 4a). Conversely, no significant change over this time period was observed in the wait-list control group (Table 2). HbA1c did not change significantly between 6 and 12 months in either group (Table 3). Between-group analyses revealed no significant differences in HbA1c at any time point (Table 4). As shown in Fig. 4b, TIR increased significantly from baseline to 12 months within the intervention group (46.6% to 56.9%, *P* = 0.002) but not within the wait-list control group. TBR did not change significantly over this time period in either group (Table 2), and no between-group differences were observed at any time point (Tables 3 and 4). There were no significant differences in change from baseline to 12 months, nor from 6 months to 12 months, between the intervention and wait-list control groups for any of the glycaemic control variables.

### Diabetes treatment satisfaction and quality of life

DTSQ scores did not change significantly within either group over time (Table 2). The between-group analysis showed a higher total DTSQ score in the intervention group at 6 months compared with controls (*P* = 0.035), while no such difference was present at 12 months (Table 4). Physical, emotional, and social health, quality of life, and perceived burden did not change significantly from baseline to 12 months within either group (Table 2). However, in the wait-list control group, social burden decreased significantly from baseline to 12 months (*P* = 0.030). The between-group analysis (Table 4) showed that the intervention group reported lower physical burden at 6 months compared with controls (*P* = 0.034), but this difference did not persist at 12 months.

The change in social burden from baseline to 12 months differed significantly between the intervention and wait-list control groups (*P* = 0.028; Table 2), with a mean (SD) change of 1.23 (6.77) points in the intervention group and − 4.26 (9.02) points in the wait-list control group. This was the only variable for which a significant difference between the intervention and wait-list control groups was observed for either change from baseline to 12 months or change from 6 months to 12 months.

### Perceived hyperglycaemia and hypoglycaemia

Perceived hyperglycaemia and hypoglycaemia did not change significantly within either group from baseline to 12 months (Table 2) or 6 months to 12 months (Table 3). Between group analyses revealed no significant differences in perceived hyperglycaemia and hypoglycaemia at any time point (Table 4).

**Table 2:** Primary and secondary outcomes at baseline and 12 months for individuals with measurements at both time points

Variable		Baseline		12 months				Change	
	Group	*n*	Mean (SD)	*n*	Mean (SD)	*P*-value^a^	*n*	Mean (SD)	*P*-value^d^
HbA1c (mmol/mol)	Intervention	33	62.8 (14.9)	33	58.6 (12.4)	0.051^b^	33	-4.24 (12.05)	0.310^e^
	Wait-list control	37	60.1 (11.4)	37	58.5 (10.9)	0.322^b^	37	-1.57 (9.50)	
Time in range (%)	Intervention	28	46.6 (14.1)	28	56.9 (16.3)	**0.002** ^b^	28	10.21 (16.21)	0.185^e^
	Wait-list control	34	50.9 (17.8)	34	55.3 (17.2)	0.159^b^	34	4.41 (17.83)	
Time below range (%)	Intervention	28	7.0 (8.2)	28	5.3 (5.3)	0.282^b^	28	-1.79 (8.61)	0.547^e^
	Wait-list control	34	5.6 (4.5)	34	4.9 (5.1)	0.423^b^	34	-0.68 (4.86)	
DTSQ, hyper (points)	Intervention	23	2.5 (1.4)	23	2.8 (1.4)	0.356^c^	23	0.30 (1.29)	0.119^f^
	Wait-list control	27	3.6 (1.3)	27	3.1 (1.7)	0.172^c^	27	-0.48 (1.76)	
DTSQ, hypo (points)	Intervention	23	1.6 (1.1)	23	1.5 (0.9)	0.644^c^	23	-0.13 (1.01)	0.602^f^
	Wait-list control	27	1.7 (1.3)	27	1.9 (1.3)	0.788^c^	27	0.15 (1.41)	
DTSQ, score (points)	Intervention	20	29.6 (4.5)	20	30.4 (4.5)	0.326^c^	20	0.80 (3.47)	0.456^f^
	Wait-list control	24	28.9 (4.2)	24	30.4 (4.1)	0.246^c^	24	1.50 (5.05)	
Physical health (points)	Intervention	24	71.1 (16.1)	24	71.5 (19.2)	0.753^c^	24	0.33 (9.17)	0.691^f^
	Wait-list control	27	68.1 (17.2)	27	71.1 (15.0)	0.474^c^	27	2.96 (18.85)	
Physical burden (points)	Intervention	24	10.5 (9.3)	24	6.1 (14.9)	0.108^c^	24	-4.33 (10.89)	0.602^f^
	Wait-list control	27	9.1 (7.8)	27	8.5 (13.3)	0.346^c^	27	-0.63 (13.00)	
Emotional health (points)	Intervention	23	71.1 (21.3)	23	72.7 (16.7)	0.833^c^	23	1.67 (17.15)	0.188^f^
	Wait-list control	27	63.8 (15.6)	27	70.4 (17.4)	0.062^c^	27	6.59 (20.55)	
Emotional burden (points)	Intervention	23	9.4 (8.7)	23	7.8 (10.8)	0.687^c^	23	-1.57 (11.94)	0.506^f^
	Wait-list control	27	13.1(10.3)	27	10.7 (14.7)	0.180^c^	27	2.44 (18.85)	
Social health (points)	Intervention	22	81.6(14.2)	22	78.1 (18.2)	0.252^c^	22	-3.50 (13.00)	0.952^f^
	Wait-list control	27	84.7 (14.5)	27	83.7 (13.0)	0.493^c^	27	-1.07 (14.64)	
Social burden (points)	Intervention	22	3.2 (7.7)	22	4.5 (9.6)	0.505^c^	22	1.23 (6.77)	**0.028** ^f^
	Wait-list control	27	4.3 (8.2)	27	0.03 (5.6)	**0.030** ^c^	27	-4.26 (9.02)	
Quality of life (points)	Intervention	23	75.7 (13.8)	23	76.2 (16.0)	0.501^c^	23	0.48 (12.26)	0.653^f^
	Wait-list control	27	76.1 (12.7)	27	75.5 (14.1)	0.903^c^	27	-0.67 (15.60)	
Burden of quality of life (points)	Intervention	22	9.8 (7.1)	22	7.0 (9.3)	0.469^c^	22	2.82 (12.19)	0.793^f^
	Wait-list control	27	9.8 (9.5)	27	8.9 (8.6)	0.389^c^	27	-0.85 (9.64)	

Note: DTSQ, Diabetes Treatment Satisfaction Questionnaire; SD, standard deviation. Significant *P*-values are given in **bold**. ^a^ *P*-value for test of within-group differences between baseline and 12 months, using: ^b^ Welch’s paired *t*-test; ^c^ Wilcoxon signed rank test. ^d^ *P*-value for difference between intervention and wait-list control groups in change from baseline to 12 months, using: ^e^ Welch’s independent samples *t*-test; ^f^ Mann-Whitney test

**Table 3:** Primary and secondary outcomes at 6 months and 12 months for individuals with measurements at both time points

Variable	Group	6 months		12 months		*P*-value^a^	Change		*P*-value
		*n*	Mean (SD)	*n*	Mean (SD)		*n*	Mean (SD)	
HbA1c (mmol/mol)	Intervention	32	62.6 (11.3)	32	59.3 (11.7)	0.080^b^	32	-3.22 (10.05)	0.386^e^
	Wait-list control	36	60.1 (11.4)	36	58.7 (11.0)	0.219^b^	36	-1.39 (6.66)	
Time in range (%)	Intervention	26	51.8 (16.6)	26	55.2 (15.1)	0.244^b^	26	3.31 (14.15)	0.944^e^
	Wait-list control	31	53.1 (16.9)	31	56.5 (17.5)	0.196^b^	31	3.58 (15.06)	
Time below range (%)	Intervention	26	6.7 (6.8)	26	4.8 (5.2)	0.082^b^	26	-1.88 5.31	0.291^e^
	Wait-list control	31	5.1 (4.5)	31	4.7 (4.9)	0.722^b^	31	-0.35 (5.50)	
DTSQ, hyper (points)	Intervention	23	2.7 (1.3)	23	2.8 (1.4)	0.699^c^	23	0.09 (1.20)	0.231^f^
	Wait-list control	26	3.4 (1.6)	26	3.0 (1.7)	0.359^c^	26	-0.35 (1.52)	
DTSQ, hypo (points)	Intervention	23	1.7 (1.3)	23	1.5 (0.9)	0.541^c^	23	-0.22 (1.44)	0.902^f^
	Wait-list control	26	2.3 (1.3)	26	2.0 (1.2)	0.471^c^	26	-0.27 (1.66)	
DTSQ, score (points)	Intervention	21	31.3 (4.7)	21	30.5 (4.5)	0.300^c^	21	-0.81 (2.86)	0.114^f^
	Wait-list control	23	28.9 (4.6)	23	30.3 (4.2)	0.223^c^	23	1.48 (5.95)	
Physical health (points)	Intervention	23	69.3 (20.0)	23	70.5 (19.0)	0.586^c^	23	1.17 (12.86)	0.976^f^
	Wait-list control	26	69.8 (13.5)	26	71.2 (15.3)	0.456^c^	26	1.38 (18.58)	
Physical burden (points)	Intervention	23	7.0 (9.0)	23	6.1 (15.3)	0.842^c^	23	-0.91 (10.67)	0.372^f^
	Wait-list control	26	10.0 (6.3)	26	8.4 (13.6)	0.332^c^	26	-1.62 (13.36)	
Emotional health (points)	Intervention	23	80.9 (20.5)	23	72.5 (16.5)	0.872^c^	23	1.57 (21.09)	0.740^f^
	Wait-list control	26	68.7 (14.0)	26	70.8 (17.6)	0.554^c^	26	2.12 (20.80)	
Emotional burden (points)	Intervention	23	6.2 (5.9)	23	7.6 (10.9)	0.695^c^	23	1.35 (10.97)	0.125^f^
	Wait-list control	26	12.3 (10.9)	26	10.3 (14.8)	0.166^c^	26	-2.04 (15.26)	
Social health (points)	Intervention	22	80.6 (12.6)	22	76.7 (17.6)	0.303^c^	22	-3.91 (12.75)	0.480^f^
	Wait-list control	26	82.8 (14.9)	26	83.8 (13.2)	1.000^c^	26	1.00 (15.09)	
Social burden (points)	Intervention	22	2.5 (7.0)	22	4.5 (9.9)	0.135^c^	22	1.95 (6.01)	0.073^f^
	Wait-list control	26	1.8 (4.8)	26	0.04 (5.6)	0.725^c^	26	-0.85 (4.51)	
Quality of life (points)	Intervention	23	75.9 (14.1)	23	75.0 (15.3)	0.930^c^	23	-0.91 (14.44)	0.513^f^
	Wait-list control	25	78.3 (10.8)	25	76.0 (14.2)	0.277^c^	25	-2.28 (12.47)	
Burden of quality of life (points)	Intervention	23	9.7 (8.7)	23	7.0 (9.1)	0.690^c^	23	-2.70 (12.30)	0.983^f^
	Wait-list control	25	8.2 (8.6)	25	8.3 (8.4)	0.909^c^	25	0.08 (5.51)	

Note: DTSQ, Diabetes Treatment Satisfaction Questionnaire; SD, standard deviation. Significant *P*-values are given in **bold**. ^a^ *P*-Value for test of within-group differences between 6 months and 12 months, using: ^b^ Welch’s paired *t*-test; ^c^ Wilcoxon signed rank test. ^d^ *P*-value for difference between intervention and wait-list control groups in change from baseline to 12 months, using: ^e^ Welch’s independent samples *t*-test; ^f^ Mann-Whitney test

**Table 4:** Primary and secondary outcomes at baseline, 6 months, and 12 months for individuals with available measurements at one or more time points

Variable	Group	Baseline			6 months			12 months		
		*n*	Mean (SD)	*P*-value	*n*	Mean (SD)	*P*-value	*n*	Mean (SD)	*P*-value
HbA1c (mmol/mol)	Intervention	35	62.8 (14.8)	0.188^b^	33	62.8 (11.2)	0.202^b^	33	58.6 (12.4)	0.982^b^
	Wait-list control	44	58.8 (11.5)		38	59.3 (11.7)		37	58.5 (10.2)	
Time in range (%)	Intervention	33	45.9 (13.3)	0.261^b^	30	50.8 (16.0)	0.513^b^	28	56.9 (16.3)	0.721^b^
	Wait-list control	43	49.8 (16.5)		35	53.5 (17.0)		34	55.3 (17.2)	
Time below range (%)	Intervention	33	7.5 (8.0)	0.422^b^	30	7.0 (7.0)	0.176^b^	28	5.3 (5.3)	0.801^b^
	Wait-list control	43	6.2 (5.8)		35	5.0 (4.3)		34	4.9 (5.1)	
DTSQ, hyper (points)	Intervention	35	2.9 (1.4)	0.062^c^	30	3.0 (1.2)	0.781^c^	23	2.8 (1.4)	0.682^c^
	Wait-list control	44	3.5 (1.4)		37	3.1 (1.6)		27	3.1 (1.7)	
DTSQ, hypo (points)	Intervention	35	1.7 (1.2)	0.438^c^	30	1.7 (1.3)	0.354^c^	23	1.5 (0.9)	0.339^c^
	Wait-list control	44	2.0 (1.3)		37	2.1 (1.3)		27	1.9 (1.3)	
DTSQs score (points)	Intervention	34	29.8 (4.3)	0.184^c^	30	30.5 (4.7)	**0.035** ^c^	21	30.5 (4.5)	0.873^c^
	Wait-list control	44	28.6 (3.8)		37	28.8 (3.8)		24	30.4 (4.1)	
Physical health (points)	Intervention	35	67.5 (17.0)	0.910^c^	30	68.3 (19.0)	0.844^c^	24	71.5 (19.2)	0.705^c^
	Wait-list control	44	66.1 (18.1)		37	69.7 (14.1)		27	71.1 (15.0)	
Physical burden (points)	Intervention	35	10.7 (8.6)	0.271^c^	30	7.3 (9.2)	**0.034** ^c^	24	6.1 (14.9)	0.488^c^
	Wait-list control	44	9.2 (10.0)		37	11.1 (8.1)		27	8.5 (13.3)	
Emotional health (points)	Intervention	34	68.2 (20.0)	0.203^c^	30	69.6 (19.3)	0.889^c^	24	73.4 (16.7)	0.446^c^
	Wait-list control	44	63.5 (17.1)		37	70.1 (15.9)		27	70.4 (17.4)	
Emotional burden (points)	Intervention	34	9.5 (8.6)	0.085^c^	30	6.9 (7.1)	0.283^c^	24	7.5 (10.6)	0.296^c^
	Wait-list control	44	13.8 (10.8)		37	10.7 (10.9)		27	10.1 (14.7)	
Social health (points)	Intervention	34	80.1 (15.6)	0.185^c^	30	78.1 (14.2)	0.056^c^	23	77.7 (17.8)	0.248^c^
	Wait-list control	44	82.9 (18.9)		37	84.4 (14.5)		27	83.7 (13.0)	
Social burden (points)	Intervention	34	4.0 (8.4)	0.365^c^	30	3.7 (8.1)	0.210^c^	23	4.3 (9.4)	0.130^c^
	Wait-list control	44	5.9 (10.2)		37	1.4 (4.1)		27	0.03 (0.0)	
Quality of life (points)	Intervention	33	73.6 (15.1)	0.859^c^	30	75.5 (14.3)	0.608^c^	24	76.0 (15.7)	0.820^c^
	Wait-list control	44	74.4 (15.6)		36	78.2 (13.5)		27	75.5 (14.1)	
Burden of quality of life (points)	Intervention	32	10.9 (8.2)	0.837^c^	30	10.4 (8.6)	0.327^c^	24	6.8 (9.0)	0.738^c^
	Wait-list control	43	10.9 (10.1)		36	8.4 (8.5)		27	9.0 (8.6)	

Note: DTSQ, Diabetes Treatment Satisfaction Questionnaire; SD, standard deviation. Significant *P*-values are given in **bold**. ^a^ *P*-value for test of differences between intervention and control groups, using: ^b^ Welch’s independent samples *t*-test; ^c^ Mann-Whitney test. The number of participants (n) is reported for each analysis and may vary across outcomes due to missing data

## Discussion

In this study, we investigated the effect of a virtual diabetes clinic for young adults with type 1 diabetes, followed over a 12-month period. All participants were offered access to the intervention as part of the study design; however, the timing differed between groups. The intervention group received access to the virtual platform throughout the entire study period (baseline to 12 months), whereas the wait-list control group was granted access only during the latter half of the study (months 6–12). Participants in the intervention group showed a reduction in HbA1c (*P* = 0.051) and a significant increase in TIR (*P* = 0.002). While no statistically significant improvements were observed in DTSQ scores or physical burden, the direction of change was maintained, suggesting some persistence of effects. During their intervention period (months 6–12), the wait-list control group showed smaller improvements in both primary and secondary outcomes compared with the changes observed in the intervention group during the initial phase (months 0–6). This difference may reflect the delayed start, reduced engagement over time, or contextual factors such as staff fatigue during the COVID-19 pandemic. Additionally, increased attention and support early in the study may have enhanced outcomes independent of the intervention itself (i.e., a Hawthorne effect) [25].

### Results in perspective

The approximately 10%-point improvement in TIR observed in the intervention group is clinically relevant, as even modest increases in TIR have been associated with a reduced risk of both microvascular and macrovascular complications in individuals with type 1 diabetes [26]. The observed reduction in HbA1c is consistent with findings from a previously published study evaluating virtual care in a similar population [18].

Engagement with the virtual diabetes care tools was higher in the intervention group, where the majority of participants used the system regularly; they primarily used the chat function, while videoconferencing was used more sparingly. This suggests that simple, easily accessible communication may be a key to enhancing patient engagement. To enable analysis of the low usage, future studies should collect information on the reasons for contact, as this could help clarify whether these or other factors affect the use of virtual diabetes care. However, the level of use observed in this study aligns with the actual utilization at the diabetes clinic during the same time period. A systematic review [6] found that technological diabetes self-management interventions using a two-way communication system providing tailored support and individual feedback is an effective strategy to support individuals with diabetes. Also, simply knowing that support is available can influence self-care behaviours even without active use [27]. The limited use of videoconferencing in our study may have attenuated the potential effect of the intervention, and future studies should investigate how different modes of communication influence both utilization and clinical outcomes. Baseline differences in educational level, with a higher proportion of less-educated participants in the intervention group, may also have influenced both usage and outcomes, and should therefore be taken into account when interpreting the results.

DTSQ scores in the intervention group showed improvement over time that appeared to be maintained at the 12-month follow-up. The wait-list control group partially caught up after gaining access to the virtual diabetes platform. A similar pattern was observed for physical burden, with participants in the intervention group reporting lower perceived burden at 6 months. At the 12-month follow-up, this reduction was less pronounced. Neither general quality of life nor social and emotional health was significantly affected by the intervention. This is consistent with findings from a previously published randomized controlled trial [28], where no significant changes in quality of life were observed among young adults. In contrast, previous studies by Raymond et al. have demonstrated that several virtual care interventions can improve diabetes distress, self-efficacy, and quality of life [7–9]. These discrepancies suggests that the psychosocial impact of virtual care may depend on the intensity, structure, and specific components of the intervention, and that the relatively low use of interactive features in the present study may have limited its effect on these outcomes. Taken together, these findings suggest that while virtual diabetes care can yield short-term improvements in perceived burden, broader psychosocial effects may depend on sustained engagement or more interactive components. At the same time, the modest changes in HbA1c observed here and in previous studies [7–9] indicate that clinical outcomes may require longer-term exposure or more intensive intervention strategies and may also require adaptation for minority groups and individuals from diverse cultural backgrounds [29, 30].

Emerging adulthood (18–25 years) involves growing independence and life transitions [31]. However, young adults with type 1 diabetes must simultaneously manage a demanding chronic illness [4], often with reduced or inconsistent healthcare engagement [32]. Consistent with work by Raymond et al. [3, 7–9], providing virtual care as a complement to standard care may bridge the gap between infrequent clinic visits, enabling young adults to get timely advice on insulin adjustments as well as sick-day management without waiting for their next appointment.

### Strengths and limitations

A strength of this study is its randomized wait-list controlled design, which increases the likelihood of group comparability at baseline. Nevertheless, the results presented above should be interpreted in light of several limitations. The study was originally planned to include 100 participants, and randomization was prepared accordingly. Recruitment started in September 2019, but from early 2020 was substantially hampered by the COVID-19 pandemic, and recruitment was therefore stopped in February 2021. Although 81 participants were initially considered to be enrolled, two randomization envelopes were missing, resulting in a final sample of 79 participants and unequal group sizes. In retrospect, extending the recruitment to achieve more balanced groups might have strengthened the study. The small sample size was a major limitation. A further limitation was the handling of missing data by listwise deletion, which assumes that data are missing completely at random (MCAR). However, the small sample size and lack of reliable proxy variables implied that formal imputation methods were not feasible. During the COVID-19 pandemic (2019–2022), Sweden adopted a comparatively less restrictive public health strategy than many other countries. At the study site, outpatient diabetes care continued to be delivered primarily in person, and the control group did not receive video meetings as part of usual care. Study follow-up assessments at 6 and 12 months were planned as in-person visits; however, some participants were reluctant to attend during the pandemic, contributing to higher-than-anticipated attrition.

A wait-list study design provided ethical advantages by ensuring that all participants had the opportunity to access the virtual care intervention. However, a wait-list design also has limitations, as it may be difficult to disentangle the true effects of the intervention when the two groups differ in their conditions and contextual factors at study initiation. It should also be noted that 18 participants did not complete the 12-month questionnaire, and so their values for HbA1c, TIR, and TBR were taken from a time point near the 12-month assessment.

The DTSQ questionnaire has several limitations, particularly related to ceiling and floor effects. The ceiling effect implies that when participants report high scores at baseline, the potential to detect meaningful improvements at follow-up is constrained. Conversely, the floor effect may be relevant for the questions on hyperglycaemia and hypoglycaemia, where low baseline scores reduce the ability to identify further decreases at follow-up, thereby limiting the measure’s sensitivity to change.

The generalizability of the findings in this study must be interpreted in light of the specific characteristics of the Swedish healthcare system. In Sweden, healthcare is predominantly publicly funded but geographically fragmented into 21 different independent healthcare regions, and access to diabetes technologies such as CGM is largely determined by national and regional reimbursement policies rather than individual financial capacity. However, CGM use among young adults is exceptionally high. Conducting the study at a single clinic may limit the generalizability of the results. Future studies including multiple clinics and more diverse populations are needed to confirm these findings.

## Conclusions

By combining virtual tools with standard outpatient follow-up, clinics can provide more flexible, accessible, and person-centred care, potentially improving long-term engagement and satisfaction among young adults with type 1 diabetes. In this context, the following components are central to integrating virtual tools with standard outpatient care:

- Maintaining a secure chat function for rapid, real-time questions and guidance.
- Providing automated or follow-up reminders to encourage adherence and continued engagement.
- Training staff in virtual communication strategies to optimize responsiveness and person-centred support.
- Integrating virtual tools with standard clinic visits to create a flexible hybrid model, accommodating both in-person and remote interactions.

Implementing these strategies may enhance accessibility, engagement, and long-term support for young adults with type 1 diabetes, while complementing standard outpatient follow-up.

## Data Availability

The datasets generated and/or analysed during the current study are not publicly available due to personal data protection legislation, but are available from the corresponding author on reasonable request.
